# Quantitate evaluation of photogrammetry with CT scanning for orbital defect

**DOI:** 10.1038/s41598-024-53826-2

**Published:** 2024-02-07

**Authors:** Arushi Beri, Sweta Kale Pisulkar, Balaji Paikrao, Ashutosh Bagde, Akansha Bansod, Akshay Shrivastava, Ritul Jain

**Affiliations:** 1 Department of Prosthodontics, Crown and Bridge, Sharad Pawar Dental College and Hospital, Datta Meghe Institute of Higher Education and Research, Sawangi (Meghe), Wardha, Maharashtra 442001 India; 2 Jawaharlal Nehru Medical College, Datta Meghe Institute of Higher Education and Research, Wardha, M.S. 44200 India; 3 Department of Orthodontics, Kalinga Institute of Dental Sciences (DU), Bhubaneshwar, Odisha India

**Keywords:** Photogrammetry, CT scanning, Orbital defects, Digitalization, Biotechnology, Optics and photonics

## Abstract

Facial deformities can be caused by cancer, tumours, trauma, infections, congenital or acquired defects and may lead to alteration in basic functions such as communication, breathing, and mastication and aesthetic thereby affecting quality of life. Traditional processes for manufacturing maxillofacial prostheses involve complicated, time-consuming and tedious processes for the patient and the operator. Impression of the defect area, which is the one of the crucial step in fabrication of prosthesis, is the longest and most difficult process as it requires a long contact with the patient. The digital revolution is now changing the landscape of prosthetic production and making the impression making procedure simpler. Digital technology reduces patient chair side time by providing more accurate display data in less time (3–5 min) than traditional methods. Digital impressions eliminate the need for bulky impression materials and provide a more comfortable patient experience.

## Introduction

Rehabilitation of various facial defects caused due to congenital or acquired due to trauma, cancer, infections, requires an external aid referred to as a prosthesis to improve functionality, aesthetics, and enhancement in quality of patient life^1^. Extremal maxillofacial prosthesis provides an improvisation in the functionality of the cranium, nose, ear, orbital, chin, maxilla and mandible^2^. Patient-specific prosthesis manufacturing is always perplexing and complex and proportional to defect complexity level. The general procedure for prosthesis manufacturing includes the impression of the defective area referred to as a negative impression which can be then used to replicate the true positive anatomy of the patients defect. The challenge in this conventional process are time-consuming, troublesome for patients and chairside technicians, inaccuracies due to deformation of soft tissue caused by weight of impression material or patient reflex movement^2–5^. In addition to these mentioned challenges, some other difficulties like matching of iris position with respect to another eye, for an orbital prosthesis is a challenge^6^. It emphasizes a clinical need for evolving accurate, less invasive, reproducible, replicable, easy in use methods for both paramedical staff and patients to capture the defect.

In order to overcome the disadvantages associated with conventional prosthesis manufacturing researchers are now looking towards the alternative technology based on three-dimensional imaging techniques like Computerized Tomography (CT-Scan)^7,8^ and Magnetic Resonance Imaging (MRI)^9^ used to capture the true three-dimensional anatomical details of the defect. Based on these details the prosthesis can be manufactured either digitally using advanced computer-aided design (CAD) tools or with the conventional approach. However, the main disadvantage with this method is the exposure of patients to hazardous radiation. Hence, researchers are now looking toward an alternative technology like non-contact three-dimensional (3D) digitization systems like laser or optical scanning and stereo-photogrammetry^11–13^. These unique techniques proved to be more useful for three-dimensional model creation of artifacts and in biomedical it has been found useful for non-defective model creation^14,15^. The major concern for this is the associated cost of hardware and proficient skill for operation. Although there are some researchers who are working on the development of a low-cost smartphone-based multi-view photogrammetry method to digitize the facial defect. But the quality of outcome is always an issue. There are some researchers who tried to digitize the facial defect with Autodesk 123d catch software but the result was not quantitatively evaluated. It seems that the focus of the photogrammetry approach is limited to digitalization and three-dimensional model creation^16–18^.

The proposed study deals with using the programmatic approach for digitization of ocular defects along with a quantitative evaluation with a gold standard method like CT scan, white structured light to find its metric accuracy. The authors also tried to quantify the effect of different capturing tools like DSLR (Full form) from company Nikon D5300 and smartphone device Google Pixel 2XL, IPhone 13 pro on the quality of three-dimensional model created based on parameters like time, accuracy, completeness, and resolution.

## Material and methods

### The object of study

**Figure 1:**
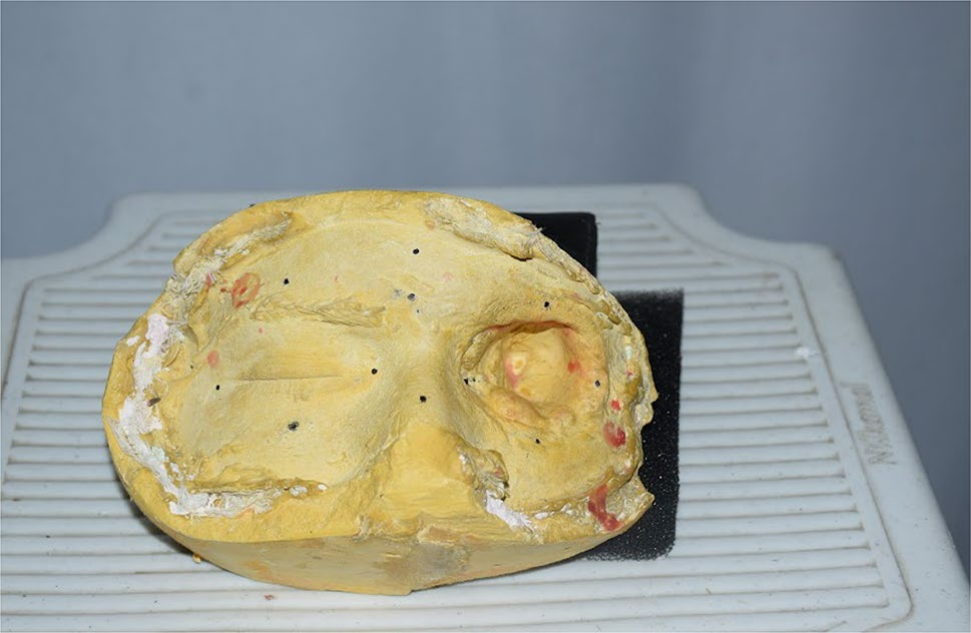
Gypsum model of maxillofacial defect.

The study was performed in gypsum model of maxillofacial defects as shown in Fig. 1. The gypsum model was obtained by the conventional method of taking an impression. As the area of interest of the study were intact eye and orbital defects. So, this part of the face is only considered at the time of taking impression conventionally while another part like the area below the nose is excluded. The approval for the study is taken from institutional ethics committee (Institutional ethical committee of Data Meghe Institute of Higher Education and Research with IEC No. DMIMS(DU)/IEC 2022/781 and and registered in Clinical Trial registry of India, CTRI Number CTRI/2022/08/044524 on date 01/08/2022. All methods were carried out in accordance with relevant guidelines and regulations and informed consent was obtained from all subjects and their legal guardian(s).

### Data acquisition and processing

Two different methods that are 3d scanning with white structured light and photogrammetry were used in acquiring the 3d data of the gypsum model.

**Figure 2:**
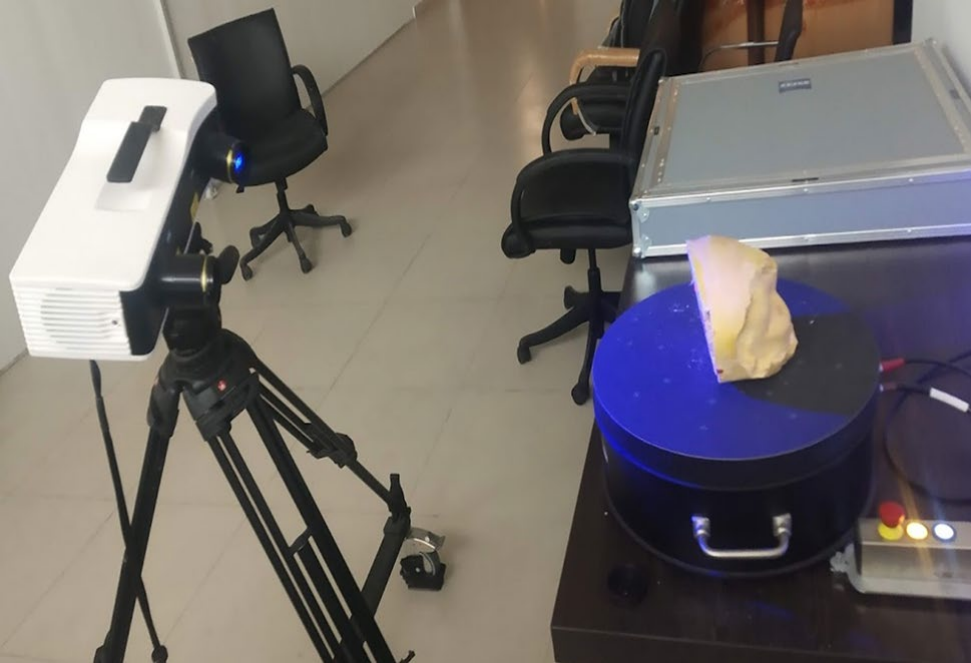
Zeiss (Comet 3D) scanner setup.

Zeiss scanner whose was employed as white structured light for creating 3d model. The setup for data acquisition with 3d scanner is shown in Fig. 2, the gypsum model was placed over the turntable, and scans were acquired at 8 angular steps. Once the scans were acquired these scans were aligned automatically in scanner software. The generated 3d models were then further processed into the software for removing noise and closing the holes and the final 3d models were exported in STL format.

**Figure 3:**
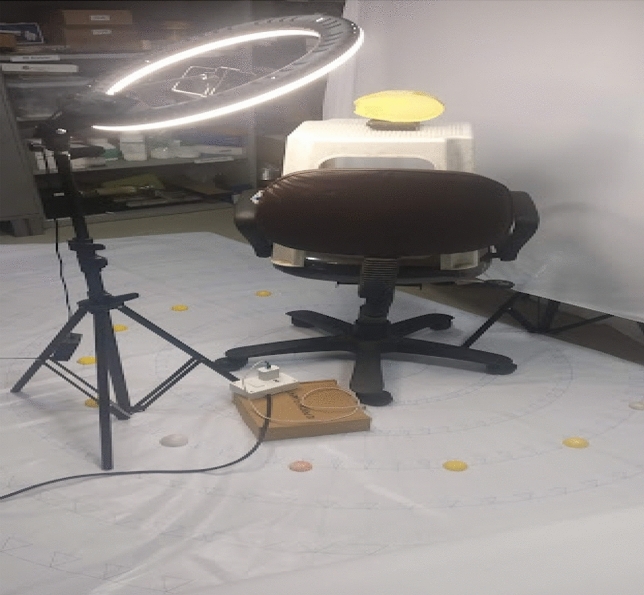
Setup for photogrammetry data acquisition.

The photogrammetry method is based on structure from a motion algorithm where multiple high images taken from different locations are converted into 3d model (Sapirstein, 2016). Nikon d5300 DSLR, and Google pixel 2xl were used for capturing High-quality 2d images of models,. Nikon 5300 DSLR is dedicated to photography itself and to acquire quality images one needs to have some understanding about operating DSLR whereas the other Google pixel 2xl is smartphone which is easy to use for non-professionals. The setup for obtaining the photographs of the gypsum model is shown in Fig. 3.

**Figure 4:**
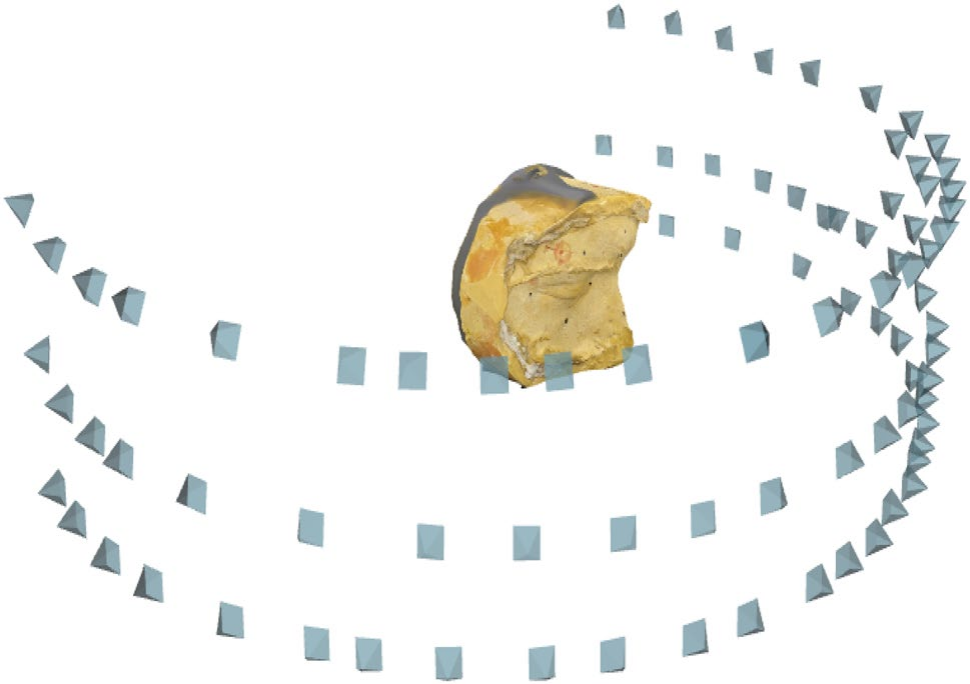
Camera positions for photogrammetry.

The models were placed on the turntable and a continuous source of light is used for producing an evenly distributed light on the gypsum model. Precaution has been taken to avoid the shadows on the object. The devices for image acquisition were attached to the tripod for reducing the motion blur. To acquire every detail of the defect area of the gypsum model, photographs were taken from three different elevations directing towards the lower, mid, and upper area of the defect, camera positions are shown in Fig. 4.A photograph number calibration study was performed to find out the effect of a number of photos on the quality of details of 3d model obtained. 90p, 60p, and 30p were taken for each gypsum model for photograph calibration study.

Autodesk Recap photo as photogrammetry software was used for creating the 3d mesh models of each gypsum model. Autodesk recap is web-based software so the user has to upload only images and other procedures will be done by software only and the final 3d model will be available. The generated 3d models were then further processed into the software itself for removing noise and closing the holes. And finally, all the models were exported in high-quality stl format. The reference 3d models were obtained by CT scan method.

### Data analysis

All the 3d models generated by different methods described in the previous section were evaluated on 4 parameters which are total time (Acquisition time + Processing time), accuracy, completeness, and resolution.

Acquisition time is defined as the time required to take scans or images. Processing time is considered as the time required to the alignment of the scan to final 3d model creation. The accuracy of all the model was calculated against the gold standard CT scanned model. Accuracy is defined as the surface to the surface distance calculated at each point of 3d mesh model. Surface to surface comparisons were performed in cloud compare opensource software. 3d mesh models of reference 3d model and model generated by other study method were imported in to Cloud Compare (CC) environment. First the two models were aligned manually by 3-point method and then fine registration is done by using ICP option available in CC. Once the 3d model aligned completely then cloud to cloud comparison was done to found the surface deviation. Cloud to mesh method is used for surface comparison as it is robust compared to other. All the further data processing done in Cloud compare software.

Completeness of the 3d models is defined as the percentage number of points below 2 mm. This is also found by using the result obtained in cloud-to mesh comparison.

Resolution is considered as no of polygon per unit area of 3d model generated by each method.

## Results

Results for all the parameters are shown in Table 1 and discussed in details below.

**Table 1:** Results obtained in different devices.

Device name	Time	Accuracy		Completeness	Resolution
		RMS	Maximum deviation defect		
DSLR 90p	3 0.2 + 135 min	0 0.55	1.32	97.67	13
DSLR 60p	1.7 + 72 min	0.68	1.36	96.81	12.88
DSLR 30p	0.8 + 25 min	0.75	2.06	96.52	12.85
PIXEL 90p	1.8 + 85 min	0.58	2.03	96.12	9.8
PIXEL 60p	1.1 + 62 min	0.72	2.12	95.82	9.42
PIXEL 30p	0.6 + 18 min	0.77	2.33	94.23	9
COMET 3D	5.8 + 12 min	0.54	1.02	97.43	32.58

Total time required is defined as time for data acquisition and time required for processing. It is found that time required for data acquisition in Google pixel is less as compared to DSLR which is due to the fast photo capturing ability of device. Also the effect of no of photos is found more in DSLR as compared to Google pixel. The time required for focusing and capturing a photo was slightly greater in DSLR. Time required for data acquisition is found more in Comet 3D that is Ziess Scanner as compared to photogrammetry devices. In Comet scanner real-time feedback whilst scanning procedure is available to operator, so whenever any portion of part is missing then the operator need to repeat the scanning procedure and which increases the time.

## Discussion

The processing time for DSLR data is found to be more as compared to Google pixel. As the DSLR data is having more details as compared to Google pixel which consequently increases the no. of calculation for photogrammetry software. As the no of photos are increased from 30 to 90 the time required for data processing increased by four times. 3D scanner is found to be very fast in terms of processing as compared to photogrammetry. It should be noted that the processing times for the photogrammetry scans only required an operator to be present for approximately 5 min of the total processing time.

The directional deviation values were used to calculate Root Mean Square (RMS) distance for each scan (Table 1). A lower RMS value indicates the scan is of higher accuracy. The Comet 3D had a lower RMS distance (0.54 mm) compared to each of the Photogrammetry scan (Table 1). DSLR with 90 photographs nearly gives RMS value equal to COMET scanner.

**Figure 5:**
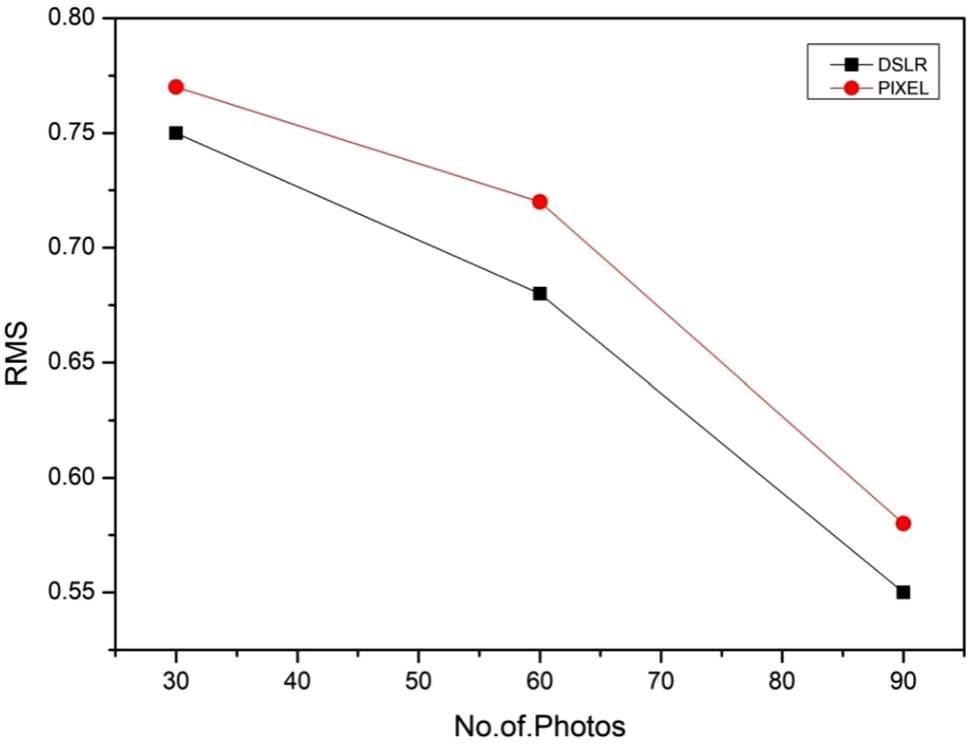
Effect of no. of photos on the RMS value.

**Figure 6:**
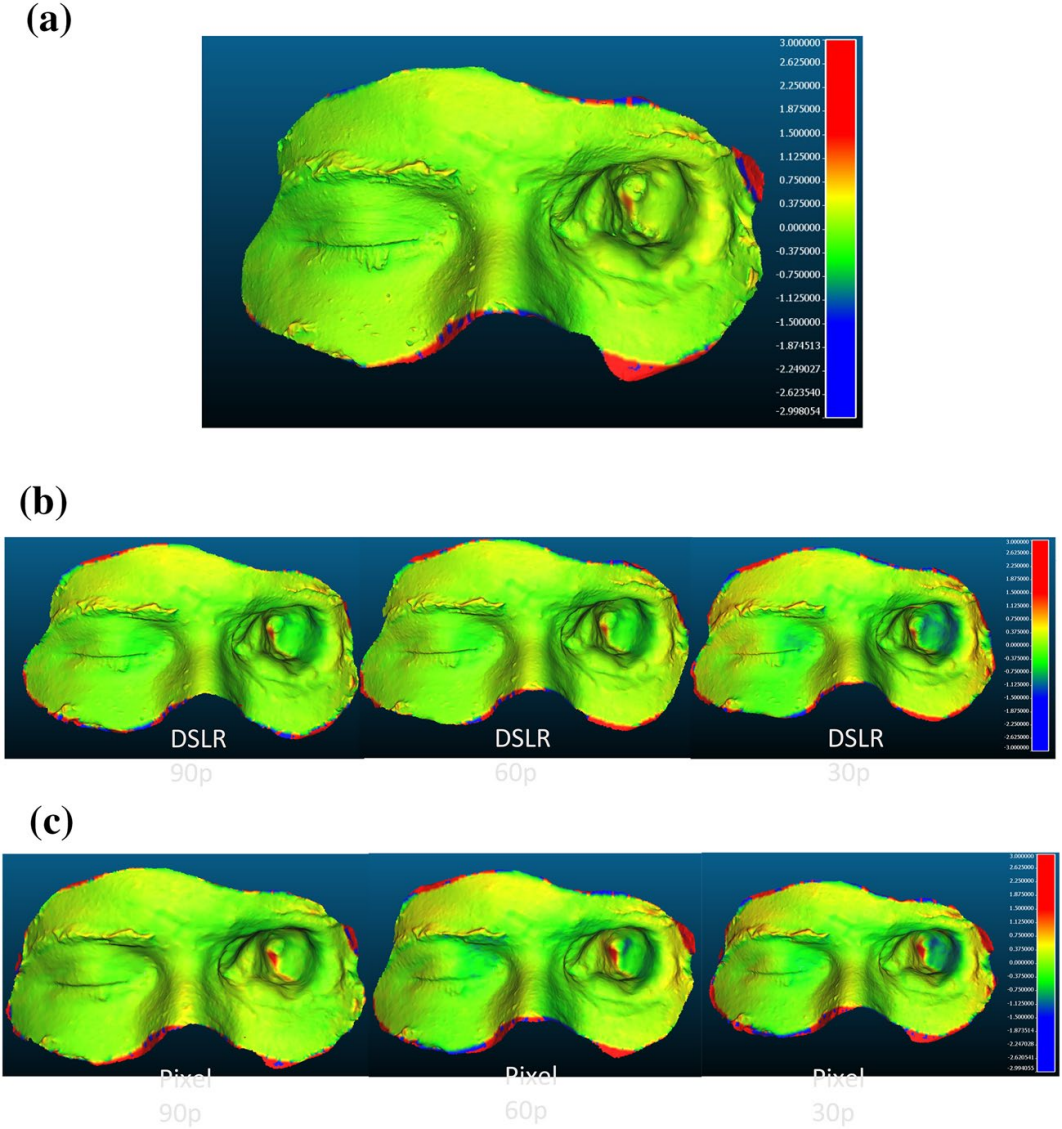
(**a**) 3D Surface deviation of Comet 3d scanned model (CT scanned model is taken as a Reference model for the analysis). (**b**) Effect of no. of photos on the 3D Surface deviation of photogrammetry model generated by DSLR data (CT scanned model is taken as a Reference model for the analysis. (**c**) Effect of no. of photos on the 3D Surface deviation of photogrammetry model generated by Google Pixel Smartphone data (CT scanned model is taken as a Reference model for the analysis.

Google pixel gives poor RMS value with 30 photographs (Fig. 5). In order to find the higher deviation areas, color map were created as shown in Fig. 6 .It is found that comet 3d scanned model has very less portion which is having deviation greater than 1 mm (shown in red color). The maximum deviation at this is found to be 1.02 mm which less than clinically significant value 2 mm. The effect of no of photos on photogrammetry model in both devices that is DSLR and Google pixel is shown in Fig. 6b and c. DSLR with 90p and 60p has shown very less area of deviation as compared to Google pixel. Maximum deviation value found in DSLR model with 90p, 60p and 30p is to be in clinically significant whereas Google pixel for 90p, 60p, and 30p has maximum deviation value greater than clinically significant. Both the devices perform purely at 30p photos.

Completeness values for all the devices found greater than 94 percentof points within 2 mm of CT scanned model for Comet 3d scanner were 98 percent. Completeness values for both the photogrammetry devices with 90 and 60 photos found to be very close to Comet 3D value. Effect of no of photographs in DSLR found to be linear whereas in Google pixel it is found that from 30 to 60 photographs the values were almost constant and for 60 to 90 p variation was linear (Fig. 6). Greater value of completeness means less no of points are having surface deviation greater than 2 mm value as compared CT scanned model. The areas which could not captured within the reliable range were the last portion of undercuts and topmost part of defect.

**Figure 7:**
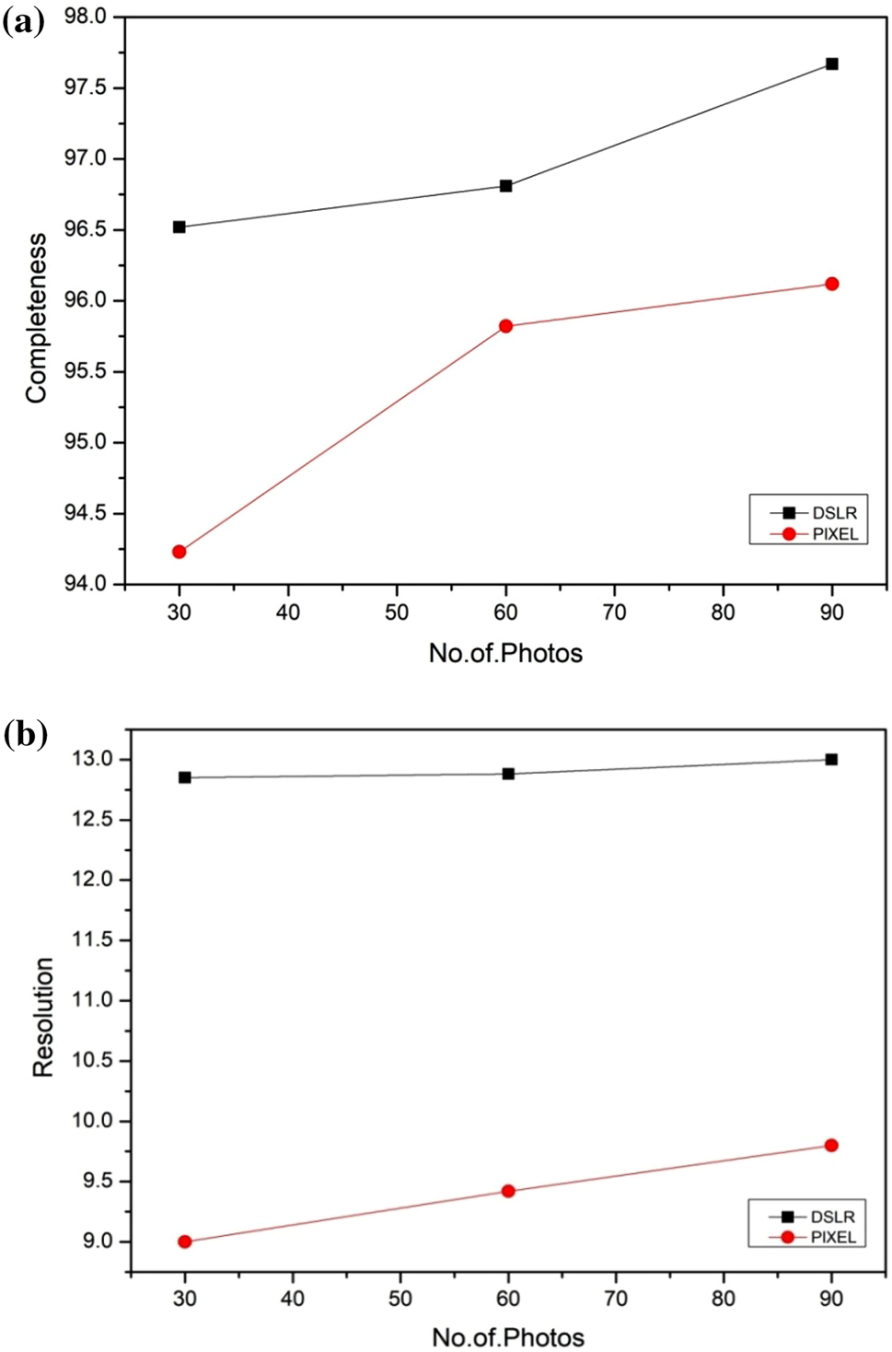
(**a**) Effect of no. of photos on the completeness value. (**b**) Effect of no. of photos on the Resolution value.

Resolution values for both the photogrammetry devices found very low as compared to Comet 3d. Comet 3d model has 32 polygons/mm2 value for resolution whereas maximum value 12 polygons/mm2 is obtained in Dsrl with 90p. There was no significant effect of no of photos found in the resolution of 3D model obtained from DSLR and google pixel (Fig. 7). As such, the aim of this study was to quantitatively evaluate the performance of photogrammetry with different devices for data acquisition as compared to high cost 3d scanning for digitization of maxillofacial defect. In this study the stone model of maxillofacial defect is digitized by using photogrammetry and 3d scanning method. The devices used for photogrammetry were Nikon D5300 DSLR and Googel pixel as smartphone. All the experimental model were compared with CT scanned model which is considered as Gold standard method. In order to evaluate photogrammetry devices suitability as alternatives to the relatively expensive 3D scanner we compared the scanning devices based on scanning and processing time, accuracy, completeness, resolution. The accuracy and completeness of each scanning device was quantified by calculating how far each point deviated from the reference scan. The accuracy and completeness values of scans generated using the Nikon D5300 DSLR (90p, 60p and 30p) and Google pixel (90p and 60p) were found to be nearly equal to those values of the scan generated by Comet 3d scanner (Figs. 5,6,7). Although The scans generated by Google pixel shown a maximum deviation values greater than the clinically significant value, the areas where it shown is away from prosthesis margin. The completeness values for all the devices found greater than 94 percentage which quiet large. In the study of effect of no of photos on the values of accuracy and completeness it is found the scanned generated by DSLR shown no such drastic changes in values as compared to Google pixel (Figs. 5 and 6).

## Conclusions

Prosthetics made using 3D scanning and modern manufacturing techniques have many advantages over those made using manual procedures. The use of technology can make procedures for patients more pleasant, boost patient customization and happiness, and cut labour and expense. However, a lot of the present literature's examples of the benefits of this technology rely on the usage of comparatively pricey 3D scanners and 3D printers. These expenses may restrict their application in clinical settings. High-quality scanning may be accomplished at a relatively low cost compared to high-end scanners because to the development of sophisticated computer software and widely accessible cellphones with high-quality cameras.

In conclusion the DSlR (60 photos) combine with photgrammetry scan produces 3d scan accurate to 1.36 mm so it can be used as alternative option for costly 3d scanning. Even though the Google pixel with 90p and 60p shows maximum value of deviation greater than the 2 mm but completeness is nearly equal to DSLR scan model the deviation area is away from the prosthesis margin, so Google pixel with 60 p can also be used as for 3d scanning of Maxillofacial defect.

## Data Availability

The datasets generated and/or analysed during the current study are not publicly available so as to keep the patients medical status confidential but are available from the corresponding author on reasonable request.
